# Re-evaluating albumin use in traumatic brain injury

**DOI:** 10.1186/s40560-025-00813-y

**Published:** 2025-08-01

**Authors:** Jean-Louis Vincent, Ricard Ferrer, Fabio S. Taccone, Christian J. Wiedermann, Peter Reinstrup

**Affiliations:** 1 Present Address: Department of Intensive Care, Erasme Hospital, Hôpital Universitaire de Bruxelles (HUB), Université Libre de Bruxelles (ULB), Brussels, Belgium; 2 Intensive Care Department, Hospital Universitari Vall d’Hebrón, Barcelona, Spain; 3 Institute of General Practice and Public Health, Claudiana College of Health Professions, Bolzano, Italy; 4 Department of Intensive and Perioperative Care, SUS University Hospital, Lund, Sweden; 5 SODIR Research Group, Vall d’Hebron Institut de Recerca, Barcelona, Spain; 6 Department of Medicine, Universitat Autonoma de Barcelona, Barcelona, Spain

**Keywords:** Albumin, Traumatic brain injury, Intracranial pressure, Outcomes

## Abstract

Traumatic brain injury (TBI) affects approximately 69 million people annually, with the majority of cases being mild-to-moderate in severity. However, in severe TBI, early management is critical and includes fluid resuscitation to control intracranial pressure (ICP) and optimize cerebral perfusion pressure. The SAFE-TBI study linked hypotonic 4% albumin to higher mortality versus saline (33.2% vs. 20.4%; RR 1.63; P = 0.003), likely due to elevated ICP, prompting guidelines favoring saline. However, these recommendations are based on low-quality evidence and overlook hyperoncotic albumin. Preclinical data confirm that hypotonicity—not albumin—drives ICP elevation. Emerging data suggest that hyperoncotic albumin (20–25%) may reduce ICP and improve outcomes. This letter highlights evidence gaps and advocates re-evaluating albumin use in TBI, especially hyperoncotic formulations.

## Letter to the Editor

Traumatic brain injury (TBI) disrupts normal brain function and is a major cause of morbidity and mortality among patients with trauma-related injuries. The global incidence of TBI is estimated at 939 cases per 100,000 people, with approximately 69 million people affected worldwide annually. Most cases of TBI are mild (81%) and moderate (11%) in severity [1]. Although most cases are mild or moderate, patients with severe TBI require intensive management to prevent secondary brain injury and optimize neurological outcomes.

Early and effective management of TBI involves prompt identification and treatment of secondary insults [2], applying a comprehensive approach that prioritizes both cerebral and systemic hemodynamic optimization. This includes reducing elevated intracranial pressure (ICP) while simultaneously ensuring adequate cerebral perfusion pressure (CPP) to prevent cerebral hypoperfusion and subsequent ischemic injury [3]. Physiological principles emphasize that fluid therapy in TBI should aim to preserve cerebral perfusion without exacerbating intracranial hypertension [4]. Achieving this balance typically requires a combination of fluid resuscitation and vasopressor therapy to maintain sufficient systemic perfusion while mitigating the risks associated with intracranial hypertension [5].

The randomized controlled SAFE trial assessed the safety of fluid resuscitation with hypotonic 4% albumin compared to normotonic, or in fact slightly hypertonic, 0.9% saline (e.g., 308 mOsm/kg) in ICU patients. Although there was no significant difference in 28-day mortality rates between treatment groups in the entire cohort, the subgroup of patients with TBI treated with 4% albumin showed a significantly higher relative risk of death than those treated with saline [6]. In a post hoc analysis, the SAFE-TBI study concluded there was a higher mortality in patients with TBI treated with 4% albumin (33.2%) compared to those treated with saline (20.4%—relative risk 1.63; P = 0.003) [7]. Considering that ICP monitoring is recommended in patients with severe TBI [8], a mechanistic study using the SAFE study data focused on patients with ICP monitoring, who represented 69.7% of the SAFE-TBI cohort, and found a significant increase in ICP and mortality in the 4% albumin group compared to the saline group during the first week. These data suggested that increased ICP is a likely mechanism for the higher mortality observed [9]. In addition, to determine whether the increased ICP was due to the hypotonicity of the 4% albumin solution or the albumin molecule itself, a preclinical Australian study compared commercially available hypotonic 4% albumin solution (4% Albumex, 278 mOsm/kg) to a novel isotonic 4% albumin solution (288 mOsm/kg) and normal saline (308 mOsm/kg) in a cross-over randomized controlled trial in sheep. The use of hypotonic albumin significantly increased ICP, whereas isotonic albumin and normal saline did not, indicating that the tonicity of the albumin solution was responsible for the increased ICP. The conclusion of this study was clear: ‘the tonicity of the albumin solution, rather than the albumin itself, is responsible for increasing ICP’ [10].

Based on the SAFE-TBI study results, the clinical practice guideline on fluid therapy in adult critically ill patients from the European Society of Intensive Care Medicine (ESICM) suggested using crystalloid isotonic saline rather than albumin for volume expansion in TBI patients [11]. However, this was a recommendation issued with low evidence, and the guideline remarks that existing randomized controlled trials compare albumin to isotonic saline, but not balanced crystalloids [11]. Balanced crystalloids are perceived as safer than saline, because they cause less metabolic acidosis [12]. Yet, Ringer’s lactate solutions are somewhat hypotonic (e.g., 273–278 mOsm/kg), which could lead to complications as with hypotonic 4% albumin [13].

Hyperoncotic albumin solutions (20–25%, typically ≥ 300 mOsm/kg, depending on formulation) have demonstrated greater effectiveness than hypotonic albumin for rapid plasma volume expansion and are widely used in various clinical settings, including liver disease, sepsis, cardiac surgery, and critical care [14]. In the context of severe TBI, hyperoncotic albumin solutions have been investigated as ICP-targeted therapy within the Lund concept. The Lund protocol, which recommends using hyperoncotic albumin at low infusion rates to maintain normovolemia and normal plasma oncotic pressure, has shown benefit in reducing or preventing increases in ICP and improving perfusion and oxygenation around contusions [15]. Ensuring normal serum albumin levels during normovolemic fluid management offers advantages such as stable hemodynamics and decreased cerebral edema by enhancing transcapillary absorption and brain perfusion [16]. Although treatment with hyperoncotic albumin has been associated with lower mortality rates [17], the evidence supporting its effectiveness in reducing mortality in patients with TBI comes from observational studies and pilot trials rather than randomized control trials. Importantly, no large randomized controlled trials have specifically evaluated the efficacy and safety of hyperoncotic albumin in TBI, leaving a significant gap in the evidence. Therefore, further evaluation is needed to address unresolved questions and research gaps.

In conclusion, current recommendations against the general use of albumin in TBI are based on a sub-study focused on hypotonic 4% albumin solution, with the quality of the evidence being very low. It is well-established that the administration of hypotonic solutions can increase ICP. Given the effects observed with hyperoncotic albumin in mitigating increased ICP in TBI patients, the current recommendation against the general use of albumin in TBI should be revised. Further research is warranted to thoroughly evaluate the potential positive effects of hyperoncotic albumin solutions in this context.

## Data Availability

No data sets were generated or analysed during the current study.

## Abbreviations

CPP: Cerebral perfusion pressure
ESICM: European Society of Intensive Care Medicine
ICP: Intracranial pressure
TBI: Traumatic brain injury
